# Modulating the Focus of Attention for Spoken Words at Encoding Affects Frontoparietal Activation for Incidental Verbal Memory

**DOI:** 10.1155/2012/579786

**Published:** 2011-11-22

**Authors:** Thomas A. Christensen, Kyle R. Almryde, Lesley J. Fidler, Julie L. Lockwood, Sharon M. Antonucci, Elena Plante

**Affiliations:** ^1^Department of Speech, Language & Hearing Sciences, The University of Arizona, Tucson, AZ 85721, USA; ^2^Department of Communicative Sciences and Disorders, New York University, NY 10012, USA

## Abstract

Attention is crucial for encoding information into memory, and current dual-process models seek to explain the roles of attention in both recollection memory and incidental-perceptual memory processes. The present study combined an incidental memory paradigm with event-related functional MRI to examine the effect of attention at encoding on the subsequent neural activation associated with unintended perceptual memory for spoken words. At encoding, we systematically varied attention levels as listeners heard a list of single English nouns. We then presented these words again in the context of a recognition task and assessed the effect of modulating attention at encoding on the BOLD responses to words that were either attended strongly, weakly, or not heard previously. MRI revealed activity in right-lateralized inferior parietal and prefrontal regions, and positive BOLD signals varied with the relative level of attention present at encoding. Temporal analysis of hemodynamic responses further showed that the time course of BOLD activity was modulated differentially by unintentionally encoded words compared to novel items. Our findings largely support current models of memory consolidation and retrieval, but they also provide fresh evidence for hemispheric differences and functional subdivisions in right frontoparietal attention networks that help shape auditory episodic recall.

## 1. Introduction 

Attention is known to alter neural processing at multiple levels of both the peripheral and central nervous systems, and both auditory and visual attention have been conceptualized as operating in both “top-down” and “bottom-up” modes [1–7]. Top-down mechanisms reflect goal-based control in order to direct attention to particular targets or to sustain attention over time. In contrast, bottom-up mechanisms have traditionally been defined by the phenomenon of reflexive attentional orienting, as when attention is drawn without intent by highly salient sensory stimuli such as a sudden loud noise or flash of light. Recently, however, some investigators have more broadly considered bottom-up effects as relevant for any incoming stimuli, with the relative saliency of the stimulus influencing whether it is ultimately encoded into memory [8]. Two recent theoretical models address the question of what roles stimulus saliency might play in first successfully encoding information into memory and then later retrieving it. As discussed below, the “Embedded Processes” model and the “Attention-to-Memory” model, while similar, also highlight the potentially divergent roles that attention at encoding may play in later recall. Brain mapping studies based on these models have only started to identify the neural substrates that underlie these cognitive processes in different domains.

In real life situations, numerous bits of information co-occur in the environment and vie for attention. Given that memory is a limited capacity system [9–11], the information encoded into memory must be restricted to what is relevant. According to the “Embedded Processes” model, attention is central to this process. Information is first encoded into working memory through an active process known as “attentional scanning” [12]. This process first searches through a set of potential memory items, then selects the most salient items and brings them into the “focus of attention” [12–14]. However, even though the attentional scanner is responsible for selecting the appropriate stimuli, its capacity is limited, and it therefore can maintain focus on only small amounts of information at a time. This also places limits on the capacity of working memory. Cowan and colleagues have speculated that regions in and around the temporal-parietal junction are key to focusing attention [14]. More recently, others have found that the majority of functional neuroimaging data on working memory is consistent with the tenets of this model [15], with ventral posterior (inferior parietal cortex and intraparietal sulcus) regions associated with attentional focus and working memory maintenance, and lateral prefrontal regions involved in the executive control of attention and further manipulation of information, once it is in working memory [16, 17]. If this interpretation is correct, then manipulations of attention at encoding should preferentially affect activation in parietal regions. 

Another recent paradigm, the “Attention-to-Memory” model [8, 18–20], shares functional features with the previous model, but it also emphasizes a distinction between the roles of ventral and dorsal parietal cortex in memory retrieval. Successful recognition memory for intentionally studied items is predicted by increased activation of *dorsal *posterior parietal cortex at stimulus encoding, whereas activation in *ventral *posterior parietal cortex at encoding predicts success in incidental memory and perceptual priming tests [21, 22]. This latter observation suggests a role for this ventral region not only in the bottom-up encoding of highly salient or unexpected sensory stimuli, but also in the successful retrieval of unintentionally encoded memories [21]. According to these prevailing theories, retrieval of incidental or perceptual memory representations requires a bottom-up shift in selective attention, whereas retrieval of intentional memories requires a top-down mechanism that reflects the individual's conscious intent to focus attention on incoming signals [21, 22]. Importantly, however, the vast majority of studies exploring the intersection of attention and episodic memory—and thus the theories on which these studies are based—are heavily grounded in the use of visual paradigms [23–30]. Even studies that focus specifically on verbal encoding have mostly employed reading rather than listening tasks [31–37]. It is therefore necessary to examine the effects of attention on item retrieval in listening tasks in order to establish whether the dorsal/ventral distinction observed in parietal cortex is specific to visual stimuli, or instead reflects a more generalized organization in this area of the brain.

To examine these ideas in greater depth, it is necessary to learn more about the role of attention in establishing a selective focus on task-relevant sensory stimuli. Moreover, in order to understand how limited attentional resources are allocated, it is also important to study the encoding of items both *within* the focus of attention as well as those* peripheral *to it. Our goal in this study was to devise a method to modulate attention toward spoken words during a simple listening task. Based on earlier studies [38], we predicted that words not intentionally within the focus of attention would be encoded into memory to some degree. During the encoding phase of our study [39], participants initially heard a list of words belonging to two semantic classes (animals or foods), and these words were presented by two different talkers (female or male). Participants were given explicit instructions to remember only the animal words presented in the female voice (the targets). Importantly, this instruction resulted in a focus on the female voice, which had the consequence of placing the nontarget food words presented in that voice also within the focus of attention. We predicted that these words would also carry higher salience at encoding than words heard in the male voice, creating what we refer to as the *High-Attention* condition in this study. Following this rationale, the food words presented in the male voice would not be fully in the primary focus of attention (the *Low-Attention* condition). We contrasted these two encoding conditions with responses to novel food words that were not previously heard at any time during the experiment. We reasoned that processing of these *NEW* words should reflect only the role of attention in initial word encoding, but not a later role in retrieval. Using this paradigm coupled with functional magnetic resonance imaging (fMRI), we were able to disambiguate the neural activation patterns associated with attention at first encoding from those reflecting the effects of attention at encoding on subsequent retrieval. 

## 2. Materials and Methods

### 2.1. Participants

We recruited 14 native English-speaking adults (9 women; ages 18–49; mean 24 years) living in the Tucson area. Exclusion criteria were a history of speech, language, or other neurological disorders, and all volunteers reported good general health with no contraindications for MRI scanning. The study was approved by the University of Arizona Institutional Review board, and written informed consent was obtained from all participants. For one participant, behavioral response data are not available due to computer error.

### 2.2. Auditory Stimuli

Word stimuli were single, concrete nouns (one to three syllables) that were either names of animals or foods. Some words were recorded in an unfamiliar female voice, and some in an unfamiliar male voice. Stimulus durations ranged from 204–903 ms (mean = 532 ms) and were presented in an event-related format with an average interstimulus interval (ISI) of 871 ± 157 ms. ISI was jittered and “null” events were included to facilitate estimation of the hemodynamic responses for deconvolution analysis (see below). Ordering of words belonging to the different stimulus categories described below was pseudorandom, and participants listened to them through MRI-compatible stereo headphones (Resonance Technology Inc., Northridge, Calif, USA).

### 2.3. Procedure

**Figure 1:**
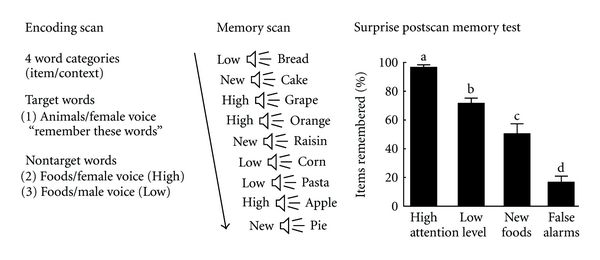
Experimental design for studying incidental encoding and memory. During the *Encoding scan* [39], listeners were presented with a list of single nouns (animals or foods). Half of the words were recorded in a female voice, and half in a male voice, thus yielding multiple combinations of item and voice context. Throughout the experiment, listeners' attention was focused on only one subset of words: animal words presented in the female voice (*Target* words). This was accomplished first by giving them explicit instructions to select and remember these words during the *Encoding scan*, and later, by instructing them to select these items by responding “yes” during the *Memory scan*. However, as predicted, some of the *Nontarget* words were also encoded unintentionally, with food words presented in the female voice carrying greater salience (the *High-Attention* condition) than food words presented in the male voice (the *Low-Attention *condition). During the *Memory scan*, participants were presented once again with both *High- *and *Low-Attention *food words, along with novel food words (*NEW*) as a control category representing the baseline attention condition. Following the *Memory scan*, a *Surprise memory test* was used to confirm the extent to which *High-Attention*, *Low-Attention*, and *NEW* food words were successfully encoded. The chart at right shows the group-averaged memory scores (*N* = 13; means ± SEM) for these three word categories, along with ten foils to measure *false alarm* rate. Bars capped by different letters are significantly different at *P* < 0.001 (two-tailed ANOVA).

The experimental design is shown in Figure 1. A prescan practice phase was followed by an encoding phase and a test phase (both with scanning). These were then followed by a surprise postscan memory test that specifically measured the listener's capacity to remember any words that were encoded during the earlier phases of the experiment, whether or not the listener was asked to remember them. The results of the test phase and scan, along with results from the postscan behavioral test, are the subject of this report. Results obtained during the encoding phase have been published previously [39].

#### 2.3.1. Encoding Phase and Scan

Participants listened to the animal and food words that were presented in either the male or female voice during a prescan practice period and during the encoding phase. Using a block design, the encoding phase consisted of two scans that were counterbalanced for order across participants. In one scan, approximately one-third of the total words used for this study were presented binaurally, with the male and female voices presented sequentially. In a second scan, two-thirds of the words were presented dichotically, with the female voice heard in one ear and the male voice heard in the other (order counterbalanced across participants). The ratio of targets (animal word + female voice) to nontargets was 1 : 3 in both tasks. Although all participants were explicitly instructed to focus on and remember the animal words presented in the female voice, these were not actually of interest for the purposes of this study. Rather, this instruction was used deliberately to assure that the primary focus of attention was on the female voice. As a result, food words presented in the female voice were associated with a higher level of attention (defined as the *High-Attention* condition) and food words presented in the male voice were associated with a lower level of attention (the *Low-Attention *condition).

#### 2.3.2. Test Phase and Scan

An incidental memory scan immediately followed the encoding scan. During this scan, participants heard words presented by a second female speaker whose voice had not been heard earlier. This precluded the possibility that the voice in the memory task could provide a cue to the encoding context. Participants were asked to respond “yes” via button press if the word presented was one of their target words (animal words originally spoken in the female voice) and “no” to all other words. Therefore, both the decision (“not a target”) and response demands (selecting the “no” button) associated with all words analyzed in this study were identical. Participants were instructed to respond quickly but accurately. During this task, participants mostly heard *nontarget* words. A combination of *High-Attention* and *Low-Attention* food words (58 trials each) as well as 58 *NEW* food words that had not been presented previously were pseudorandomized and presented in a fixed order to all participants. To lessen fatigue, participants were tested over the course of two separate scans (each lasting ~9 min with a brief rest in between), during which a combined total of 232 words (randomized with 58 nulls) were presented. Data from the two scans were then concatenated for further analysis.

#### 2.3.3. Surprise Postscan Memory Test

In order to assure that there was an effect of the attentional manipulation at encoding, we administered a surprise memory test within 15 minutes of the final scan. Participants were presented verbally with a list of 40 food items and asked to recall whether they had heard each word at any time during the experiment. The list consisted of 10 *High-Attention*, 10 *Low-Attention*, and 10 *NEW* food words that were all heard during the study phase and scan. A fourth category, 10 food items not presented in any of the scans, was added to measure false-alarm rate. These 40 words were presented verbally in a fixed pseudorandom order by the experimenter and the participants verbally responded “yes” or “no” to indicate whether they remembered having heard these words at any time during the experiment.

### 2.4. Whole-Brain fMRI Imaging

Scans were acquired with a 3.0T GE Signa VH/i scanner (General Electric Medical Systems, Madison, Wis, USA) equipped with a quad-head RF coil. First, T1-weighted, fast-spin echo (FSE) axial images covering the entire brain were acquired in 26 slices with an in-plane resolution of 3.44 × 3.44 × 5 mm. Next, two functional T2*-weighted scans were acquired using a spiral in/out pulse sequence (TR = 2.3 s, TE = 30 ms, flip angle = 90°, 26 slices at 5 mm with no gap, matrix = 64 × 64, FOV = 22 cm^2^) [40]. Finally, high-resolution spoiled gradient-echo (SPGR) images were obtained in the sagittal plane (TR = 30, TE = min, flip angle = 30°, 124 slices at 1.5 mm, matrix = 256 × 256, FOV = 25 cm^2^) and aligned with the FSE images for improved regional localization and coregistration of functional data across participants after transforming the images into Talairach space [41]. Due to scanner-related problems, we were unable to use the SPGR images from three participants, and instead relied on the first set of axial FSE anatomical images for alignment with the functional scans.

#### 2.4.1. Data Analysis

Structural and functional brain images were analyzed for each participant individually with AFNI [42] followed by a group analysis. Blood-oxygenation-level-dependent (BOLD) contrast images were coregistered with anatomical data after preprocessing using standard procedures for slice-time correction, removing linear signal drift and correcting for head motion. All volumes were realigned to the base volume and spatially smoothed using a 6 mm Gaussian kernel. Data were then normalized to a scale of 0–100%, and functional images were coregistered to the structural data followed by transformation into standard Talairach space. The first four volumes in each run were also discarded to allow for T1 equilibration, and the two runs were then concatenated for further analysis. A general linear model using a gamma-spline hemodynamic response function was used to estimate magnitude parameters for events of interest for each stimulus condition in each individual. In order to capture the amplitude of the BOLD activity over time, eight separate activity models were developed. The first was time locked to the stimulus onset, and each of the seven subsequent models was offset by one-TR (2.3 s) increments from stimulus onset. Stimulus functions were then convolved with the fMRI time-series data from each individual. Parameter estimates for the resulting regressors for each condition were calculated using the least-squares fit of the models to the time-series data. Finally, a group analysis was performed with repeated-measures ANOVA (treating individuals as a random effect) to help confirm key cortical regions that showed differential neural activity for each of the four conditions. Monte Carlo simulation (8 mm FWHM blur; 1000 iterations) was used to correct the group data for multiple comparisons. Voxel-wise (uncorrected) threshold was *P* = 0.005, and minimum corrected cluster volumes in original space were 32 contiguous voxels at *P* < 0.05.

#### 2.4.2. Data-Driven Cluster Analysis

To identify regions of interest (ROI) associated with positive BOLD signal across the *High-Attention*, *Low-Attention,* and *NEW* categories, we first combined the three group-averaged datasets. This allowed us to develop ROI that were associated with activation in any of the three conditions of interest. We were particularly interested in areas in and around the dorsolateral prefrontal cortex and the temporoparietal junction due to their proposed involvement in executive control as well as phonological short-term storage and language processing [12, 43, 44]. However, since the proposed involvement of these regions is based almost entirely on visual rather than auditory paradigms, and there is growing evidence for bilateral involvement in language processing [45, 46], we examined the following ROI in both hemispheres. Magnitude estimates and peak Talairach coordinates are listed in Table 1.

Masks corresponding to distinct anatomical regions of interest were developed, and ROI were defined based on the threshold-corrected regions of significant activation. Brain regions showing significant positive BOLD activation are listed in Table 1 and described as follows (approximate Brodmann areas in parentheses): *Frontal Lobe* (dorsal to ventral): medial frontal gyrus (6/32), middle frontal gyrus (9/46), inferior frontal gyrus (47/pars orbitalis), and anterior insular cortex (13). *Parietal Lobe* (inferior parietal lobule): angular gyrus (39) and supramarginal gyrus (40). We looked for additional ROI along the anterior-posterior axis of the superior temporal lobe due to its routine involvement in speech and language tasks [47–51], but found only one with significant activation: superior temporal gyrus including and extending around the transverse temporal gyrus (BA41/42). Sub-cortical ROI included basal ganglia (caudate body) and thalamic nuclei (anterior). Finally, several ROI were localized to the anterior and posterior lobes of the cerebellum.

## 3. Results and Discussion

### 3.1. Surprise Postscan Memory Test

The surprise memory test was intended to provide proof of concept that the attentional manipulation at encoding actually produced an effect. Specifically, *High-Attention* words should be better remembered than *Low-Attention* words, and each of these should be better attended than *NEW* words. Conversely, participants should not indicate recognition for words introduced as foils that were not presented earlier at any time in the experiment.

Figure 1 shows the result of the group memory analysis (full variance model: *F*_3,48_ = 17.44, *N* = 13 listeners; *P* < 0.001, two-tailed). As predicted, the strongest memory scores were for responses to the *High-Attention* words. These words showed the best hit rate at 93.8 ± 2.4% (mean ± SEM), compared to 70.8 ± 4.6% for *Low-Attention* words, and 51.0 ± 6.5% for *NEW* words (all means significantly different based on post-hoc, two-tailed ANOVA; see Figure 1). Additionally, words introduced as foils during the postscan memory test were correctly rejected 86.2 ± 3.3% of the time, indicating a low false alarm rate.

### 3.2. fMRI Results

**Figure 2:**
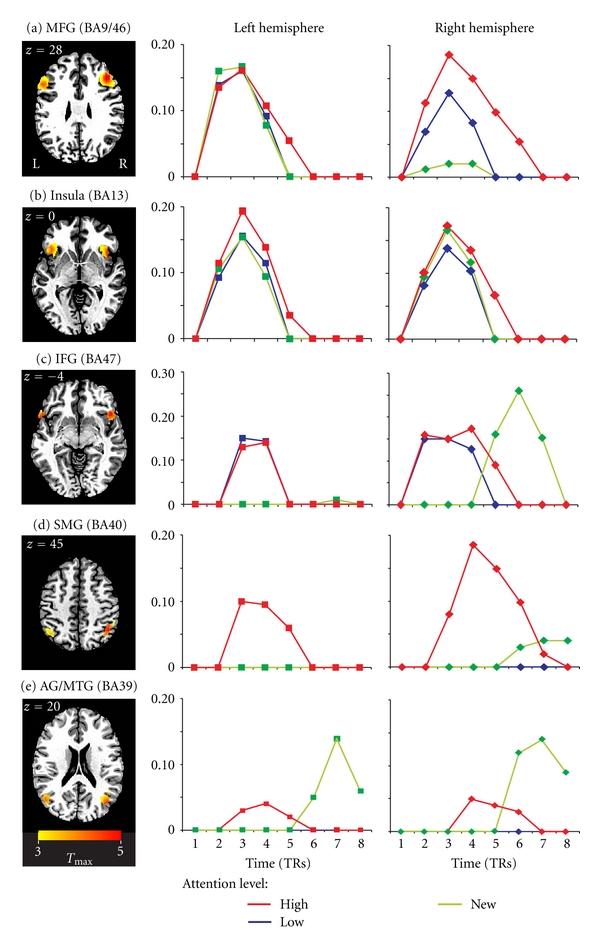
Brain regions showing significant group activation (*N* = 14) associated with recognizing previously-encoded words (*High Attention*, *Low Attention*), and *NEW* words relative to the resting state. At left, axial slices show patterns of BOLD activation in each bilateral ROI overlaid onto canonical anatomical images (*z*-depth in mm). (a) Middle Frontal Gyrus, MFG; (b) Anterior Insula; (c) Inferior Frontal Gyrus, IFG; (d) Supramarginal Gyrus, SMG; (e) Angular Gyrus/Middle Temporal Gyrus, AG/MTG. To the right of the activation maps in each row are plots of % BOLD signal change associated with memories of items in each word category, plotted over time (8 TRs). Each value represents peak activity extracted from each ROI at each time point, then averaged over the entire group. Only activity peaks that survived correction for multiple comparisons are shown. No standard errors were above 0.01%, therefore error bars were omitted for clarity.

A temporal analysis of the BOLD responses for each stimulus condition was performed by modeling the fMRI time series over a range of time lags, as shown in Figure 2. Although this analysis lacks the temporal precision of electrophysiological methods [1], it can provide valuable information about the relative timing of neural events associated with each test condition [31]. As stated above, eight separate hemodynamic response models were constructed for each stimulus type, and the peak of each model was time-shifted by one TR in order to capture peak responses across an 8-TR (18.4 s) time window. We then calculated the BOLD responses from the peak activation at each time lag, as shown in Figure 2. As expected, strong BOLD activity was observed in the superior temporal lobe in the vicinity of primary auditory cortex, and this activity did not vary with the three stimulus conditions (Table 1). In addition to the expected early activity in left superior temporal gyrus, BOLD responses associated with all three stimulus categories were, on average, 20% greater in right superior temporal gyrus, which is consistent with the other right-lateralized activation patterns discussed below. 

#### 3.2.1. Segregation of Function in Dorsal and Ventral Prefrontal Cortex 

As shown in Figure 2(a), identification of words in all three categories was associated with activity in left middle frontal gyrus (a portion of dorsolateral prefrontal cortex—DLPFC) and this activity was closely mirrored in anterior insular cortex (Figure 2(b)). Furthermore, the time course of the BOLD response in these two left-hemisphere regions was similar for all three word categories regardless of the initial encoding condition. In contrast, right DLPFC showed a distinctly different response pattern, with *High-Attention* words yielding significantly greater responses relative to *Low-Attention* words, and the latter yielding significantly greater responses relative to *NEW* words. This pattern indicates a strong differential effect of attention at encoding in right DLPFC (BA9/46) compared to left DLPFC (Figure 2(a)) and bilateral insula (Figure 2(b)). Importantly, this result is consistent with earlier studies using written words that linked activity in this region of right DLPFC with post-retrieval processing and/or monitoring functions [37, 52–54]. If right DLPFC is indeed involved selectively in post-retrieval processing, we would also expect, in accord with the “Attention-to-Memory” model, that words strongly attended at encoding might evoke a greater memory response than words only weakly attended at encoding. This graded pattern of activity was, in fact, observed for the *High-* and *Low-Attention* words, as shown in Figure 2(a). Conversely, this region would not be expected to activate in response to *NEW* words because these words were not yet encoded into memory. In accord with this hypothesis, only negligible positive BOLD activity was observed in right DLPFC in response to *NEW* words (Figure 2(a), right column). 

As shown in Figure 2(c), another bilateral set of clusters was located in the inferior frontal gyrus (IFG). This area is believed to be an important rostral component of the so-called “ventral frontoparietal stream” that is frequently observed in studies involving the detection of novel or low-frequency events, particularly when they are unexpected [3]. Although this pathway has been observed in numerous visuospatial attention studies, our findings using auditory language stimuli are also consistent with this anatomical framework. Figure 2 shows how the three stimulus conditions in our study differentially activated the ventral frontoparietal stream. The focal point for these clusters in the frontal lobes was found in pars orbitalis (BA47), and in accord with the ventral frontoparietal model, activity was also lateralized to the right hemisphere (Figure 2(c), right column). Both the *High*- and *Low-Attention* conditions were associated with comparable activation in left and right IFG. However, in left IFG, a BOLD response on a similar time course was absent for the *NEW* words, indicating that the early response in IFG to previously encoded words may specifically reflect memory for these previously presented items. Despite the absence of an early BOLD response in left IFG for *NEW* words, these items were instead associated with a late-onset response in the right hemisphere (Figure 2(c), right column). In other words, responses to the *NEW* words differed from the previously attended words both in terms of hemispheric lateralization and in the late time course of activation in IFG (BA47), suggesting that this late activation more likely reflects the initial encoding of *NEW* words into memory. 

#### 3.2.2. Segregation of Function in Ventral Posterior Cortex

With respect to language processing, converging evidence suggests a key role for posterior parietal and temporal regions in verbal memory and attention [12, 31, 37, 44]. One memory model predicts that items correctly identified as previously encountered (old) will trigger increased activity in left intraparietal cortex, relative to missed old and correctly rejected novel items [31]. However, as noted above, selective attention is another cognitive function that is commonly associated with posterior parietal cortex, and it remains unclear how the influence of attention may affect the neural networks underlying recollection and familiarity, especially in the auditory domain. 

It has been proposed that inferior parietal lobule, a region within ventral posterior parietal cortex, may serve a specialized function in the expression of attention, as proposed in both the Embedded-Processes model [14, 15] and the Attention-to-Memory (AtoM) model [8, 18–20]. These models allowed us to generate informed predictions about the neural substrates underlying a possible attention-dependent memory effect in our study. If ventral posterior parietal cortex serves specifically as the “focus of attention” as in the Embedded-Processes model, we would expect activation in this region to be differentiated according to the level of attention directed toward each stimulus at encoding, as shown in Figure 1. Another prediction is that any region responsible for the *reactivation* of focused attention prior to memory retrieval should display activity earlier in the hemodynamic response than regions responsible for post-stimulus processing steps, such as semantic analysis. 

As shown in Figure 2(d) and Table 1, the principal locus of activation in posterior parietal cortex was found bilaterally in the supramarginal gyrus (SMG). This activity, furthermore, was associated specifically with listening to and identifying the *High-Attention* words, but not the *Low-Attention* words. This is consistent with the notion that activation in and around the SMG is related to attentional scanning that placed our *High-Attention* words into the “focus of attention” according to the Embedded-Processes model. To examine the spatial location of the group activation in greater detail, the activation was overlaid onto the individual anatomical scans of each participant. This participant-by-participant anatomical localization revealed that the peak activation was located in the left SMG for 86% (12 of 14 individuals), and in the right SMG for 64% of the participants. As shown in Table 2, activation in the left hemisphere was completely isolated to the SMG in 36% of listeners, whereas in 50%, the activity also spread from the SMG into the adjoining intraparietal sulcus (IPS). In contrast, the primary focus of activation in the right hemisphere was more variable, localizing to the SMG for 50% of the participants, to the angular gyrus (AG) for 29% of participants, and to the IPS for 21% of participants. This greater variation likely reflects the greater variability of the anatomical landmarks within the posterior right hemisphere for our participants.

In contrast to these spatial activation patterns for previously encoded *High-Attention* words, the principal locus of activation in posterior parietal cortex for *NEW* words was associated with delayed activity in BA39; specifically AG and the adjacent portion of posterior middle temporal gyrus (pMTG) in both hemispheres (Figure 2(e); Table 3). In the left parietal lobe, the activity was centered within the AG for 64% of the participants (9 of 14 individuals) with additional activity that spreads around the peak activation into pMTG. In the right parietal lobe, the primary focus was centered more often in pMTG (64%) than in AG (36%) (Table 3). Thus the BOLD activation associated with *NEW* words was distinctly more ventral and posterior than the parietal activation pattern for previously encoded *High-Attention* words. Moreover, the time course of *NEW* word-related activity in right posterior parietal cortex was similar to that observed in right IFG for *NEW* words (compare Figures 2(c) and 2(e)). This finding suggests that the timing of activation in right BA39 may be an important determinant in how different stimulus contexts are represented in this region. The timing difference may reflect two distinct functional roles for this component of the frontoparietal attention network in processing previously encoded (*High-Attention*) words and newly encoded (*NEW*) words. One possibility is that the delayed response in BA39 may reflect the increased time required to access semantic content associated with these *NEW* words [55], or alternatively, activation may be associated more directly with processing novel events [3]. 

**Figure 3:**
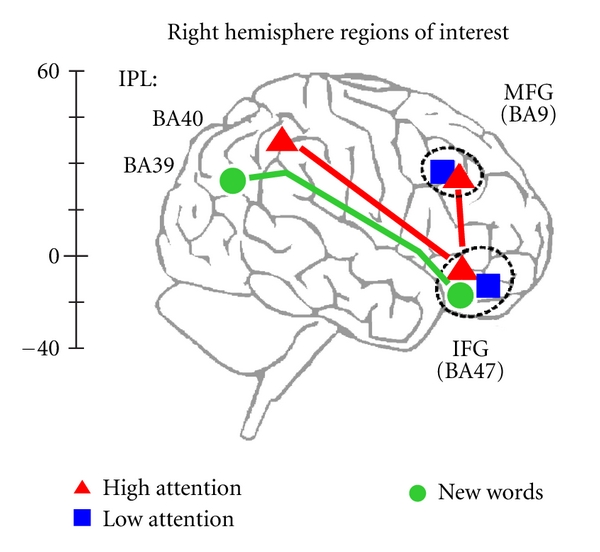
Neuroanatomical model of attentive listening networks in the right hemisphere. Both frontoparietal pathways belong to the proposed ventral, “bottom-up”, attention stream (see text). The top stream that processes *High-Attention* words (red pathway) connects BA40 in inferior parietal lobule (IPL) and BA47 in inferior frontal gyrus (IFG). The delayed-activation stream for novel stimuli (green pathway) shows the relationship between BA39 in IPL and BA47 in IFG. The red stream for processing *High-attention* words has another frontal component in right middle frontal gyrus (MFG) that appears to play a role in later postretrieval processing (see text). Responses to the *Low-attention* words were not observed in parietal cortex, but were present in BA9 and BA47, as well as in bilateral anterior insula (BA13) and dorsal anterior cingulate/supplementary motor area (BA6) (Table 1).

Based on these results, we propose a neuroanatomical framework that involves two subnetworks in the ventral frontoparietal attention stream, as shown in Figure 3. The relatively early onset activation for previously encoded words in BA40 and BA47 suggests an early attentional role for this subnetwork (Figure 3, *red pathway*), while the delayed time course of *NEW* word responses in BA39 and BA47 could reflect the activity of a separate ventral pathway associated principally with bottom-up processing of novel word stimuli (*green pathway*). Our results are therefore consistent with previous findings that ventral parietal cortex plays a pivotal role in language-based tasks [37, 55–57]. Inferior parietal activity has been found more reliably in studies of working memory than in those of explicit recall [15, 58–60], and our results are also consistent with these findings. Moreover, during the encoding phase of our study (Figure 1), right BA40 was activated at the time our spoken stimulus words were first encoded [39]. The results of the memory phase in the present study show that right BA40 was reactivated at retrieval, a finding strongly in line with the transfer-appropriate processing model [10, 25, 29–31, 61–63]. We propose that early selective activation of right BA40 serves to initiate incidental memory for the studied words by bringing them back into the “focus of attention” that was established earlier during encoding [13, 64–66].

## 4. Conclusion 

In summary, our data support a functional framework in which the brain regions that are engaged in identifying words are also sensitive to the level of attention at the time the words were initially encoded. A structural model generated from our findings is illustrated in Figure 3. Our data are consistent with a “focus of attention” centered in right SMG (BA40), but we also identified a frontal region in right DLPFC (BA9/46) that is consistent with an “Attention-to-Memory” function in postretrieval processing. Other frontal regions (left DLPFC) were insensitive to the attentional manipulation at encoding, responding similarly to both the two studied word categories as well as to the newly encoded (*NEW*) words. The pattern in left DLPFC is therefore consistent with other cognitive processes such as executive control of attention that would not be expected to vary across our word categories. A key result was that once a spoken word was unintentionally encoded, subsequent retrieval of that word varied as a function of the initial level of attention directed toward the word at the time of encoding. This is consistent with the notion that attention is critical not only for efficient encoding of words into memory, but it also helps to preserve salient information that is required for the successful retrieval of that information from memory. 

## Figures and Tables

**Table 1:** Regions selected for ROI analysis.

	Left hemisphere		Right hemisphere	
	*x*, *y*, *z*	*T*_max⁡⁡_	*x*, *y*, *z*	*T*_max⁡⁡_
Condition 1: *High Attention *				
* Frontal lobe *				
Medial frontal gyrus (6/32)	−2, 3, 48	8.97	1, 12, 45	7.31
Middle frontal gyrus (9/46)	−52, 22, 28	3.26	47, 18, 37	3.17
Inferior frontal gyrus (47)	−52, 17, −3	3.72	46, 16, −4	4.39
Insula (13)	−36, 21, 5	4.92	36, 21, 6	6.50
* Parietal lobe *				
Supramarginal gyrus (40)	−35, −62, 39^(1)^	3.76	39, −55, 46^(2)^	3.39
* Superior temporal lobe*				
Heschl's gyrus (41/42)	−50, −18, 10	5.54	53, −17, 10	9.09
* Caudate body*	−14, −6, 19	4.32	14, −6, 19	3.77
* Cerebellum—*anterior lobe	−32, −50, −27	3.84	26, −53, −26	5.59
* Cerebellum—*posterior lobe	−7, −69, −23	2.81	6, −69, −23	3.04
* Thalamus—*anterior	−7, −13, 1	5.62	1, −9, 14	4.19

Condition 2: *Low Attention *				
* Frontal lobe *				
Medial frontal gyrus (6/32)	−2, 3, 47	7.05	1, 12, 45	5.99
Middle frontal gyrus (9/46)	−50, 17, 27	3.78	53, 16, 28	2.95
Inferior frontal gyrus (47)	−50, 24, −1	3.98	49, 26, −2	3.11
Insula (13)	−34, 23, 4	4.38	40, 16, 0	4.46
* Superior temporal lobe*				
Heschl's gyrus (41/42)	−57, −21, 13	3.60	51, −18, 10	8.45
* Cerebellum—*anterior lobe	−36, −47, −27	5.89	31, −50, −27	3.69
* Cerebellum—*posterior lobe	−1, −70, −21	8.57	7, −70, −23	2.88
* Thalamus—*anterior	−8, −9, 9	4.82	5, −11, 12	5.21

Condition 3: *NEW* words				
* Frontal lobe *				
Medial frontal gyrus (6/32)	−1, 9, 46	5.94	1, 12, 45	7.31
Middle frontal gyrus (9)	−52, 19, 27	3.26	57, 22, 27	3.17
Inferior frontal gyrus (45)	−59, 22, 7	3.48	49, 26, 0	3.18
Inferior frontal gyrus (47)	—, —, —	—	51, 22, 1	2.89
Insula (13)	−42, 15, 0	3.19	42, 14, −1	6.50
* Parietal lobe*				
Angular gyrus/middle temp. gyr. (39)	−41, −63, 24	3.76	46, −65, 21	3.39
* Superior temporal lobe*				
Heschl's gyrus (41/42)	−58, −18, 11	5.54	52, −14, 10	9.09
* Cerebellum*—anterior lobe	−36, −54, −28	3.21	38, −54, −27	3.00
* Cerebellum*—posterior lobe	−1, −69, −22	2.83	5, −72, −22	2.30
* Thalamus*—anterior	−8, −8, 12	4.70	3, −8, 11	3.25

Group-averaged data showing peak intensity values (*T*_max⁡⁡_) and coordinates (mm in Talairach space) for activated clusters (closest Brodmann areas in parentheses) in each of the three conditions compared to the resting state. Corrected activation threshold = *P* < 0.05, *N* = 14. ^(1)^Activity spread into intraparietal sulcus in 58% of listeners. ^(2)^Activation in SMG, but peak was more localized to angular gyrus in 29%, and intraparietal sulcus in 21% of listeners.

**Table 2:** Regions of primary focus and secondary spread for parietal ROIs associated with the *High-Attention* condition in each of the 14 subjects.

	Left hemisphere		Right hemisphere	
	Primary focus	Secondary spread	Primary focus	Secondary spread
S1	SMG	IPS	SMG	AG
S2	SMG	—	AG	SMG/IPS
S3	SMG	IPS	SMG	IPS
S4	SPL	IPS	AG	SMG/IPS
S5	SMG	IPS	SMG	IPS
S6	SMG	IPS	SMG	AG
S7	SMG	IPS	SMG	IPS
S8	SMG	IPS	SMG	IPS
S9	IPS	SMG	IPS	SMG
S10	SMG	—	IPS	SMG
S11	SMG	—	SMG	—
S12	SMG	—	IPS	—
S13	SMG	—	AG	SMG/IPS
S14	SMG	IPS	AG	SMG/IPS

AG: angular gyrus (BA39); IPS: intraparietal sulcus (BA40/7); SMG: supramarginal gyrus (BA40); SPL: superior parietal lobule (BA7).

**Table 3:** Regions of primary focus and secondary spread for parietal ROIs associated with the *NEW* condition in each of the 14 subjects.

	Left hemisphere		Right hemisphere	
	Primary focus	Secondary spread	Primary focus	Secondary spread
S1	AG	pMTG	pMTG	—
S2	SMG	AG	pMTG	—
S3	AG	pMTG	pMTG	—
S4	AG	pMTG	pMTG	—
S5	AG	pMTG	pMTG	AG
S6	AG	pMTG	pMTG	—
S7	SMG	AG	AG	pMTG
S8	AG	pMTG	AG	pMTG
S9	AG	pMTG	pMTG	—
S10	SMG	AG	AG	—
S11	AG	pMTG	pMTG	—
S12	SMG	AG	AG	—
S13	SMG	AG	AG	pMTG
S14	AG	pMTG	pMTG	—

AG: angular gyrus (BA39); pMTG: posterior middle temporal gyrus (BA39); SMG: supramarginal gyrus (BA40).
